# The disease of corruption: views on how to fight corruption to advance 21^st^ century global health goals

**DOI:** 10.1186/s12916-016-0696-1

**Published:** 2016-09-29

**Authors:** Tim K. Mackey, Jillian Clare Kohler, William D. Savedoff, Frank Vogl, Maureen Lewis, James Sale, Joshua Michaud, Taryn Vian

**Affiliations:** 1 Department of Anesthesiology, University of California, San Diego School of Medicine, San Diego, CA USA; 2 Division of Global Public Health, University of California, San Diego School of Medicine, Department of Medicine, San Diego, CA USA; 3 Global Health Policy Institute, 6256 Greenwich Drive, Mail Code: 0172X, San Diego, CA 92122 USA; 4 WHO Collaborating Centre for Governance, Transparency and Accountability in the Pharmaceutical Sector, University of Toronto, Toronto, Ontario Canada; 5 Leslie Dan Faculty of Pharmacy, Munk School of Global Affairs, Dalla Lana School of Public Health, University of Toronto, Toronto, Ontario Canada; 6 Center for Global Development, Washington, DC, USA; 7 Transparency International, Secretariat, Berlin, Germany; 8 The Partnership for Transparency Fund, Washington, DC, USA; 9 Georgetown University, Washington, DC, USA; 10 Aceso Global, Washington, DC, USA; 11 Transparency International UK, London, UK; 12 Kaiser Family Foundation, Washington, DC, USA; 13 Johns Hopkins University School of Advanced International Studies, Washington, DC, USA; 14 Boston University School of Public Health, Boston, MA USA

**Keywords:** Global health, Corruption, Anti-corruption, Sustainable Development Goals, Good governance, International development, Global health governance

## Abstract

Corruption has been described as a disease. When corruption infiltrates global health, it can be particularly devastating, threatening hard gained improvements in human and economic development, international security, and population health. Yet, the multifaceted and complex nature of global health corruption makes it extremely difficult to tackle, despite its enormous costs, which have been estimated in the billions of dollars. In this forum article, we asked anti-corruption experts to identify key priority areas that urgently need global attention in order to advance the fight against global health corruption. The views shared by this multidisciplinary group of contributors reveal several fundamental challenges and allow us to explore potential solutions to address the unique risks posed by health-related corruption. Collectively, these perspectives also provide a roadmap that can be used in support of global health anti-corruption efforts in the post-2015 development agenda.

## Background

### Tim Mackey (Fig. 1)

**Fig. 1:**
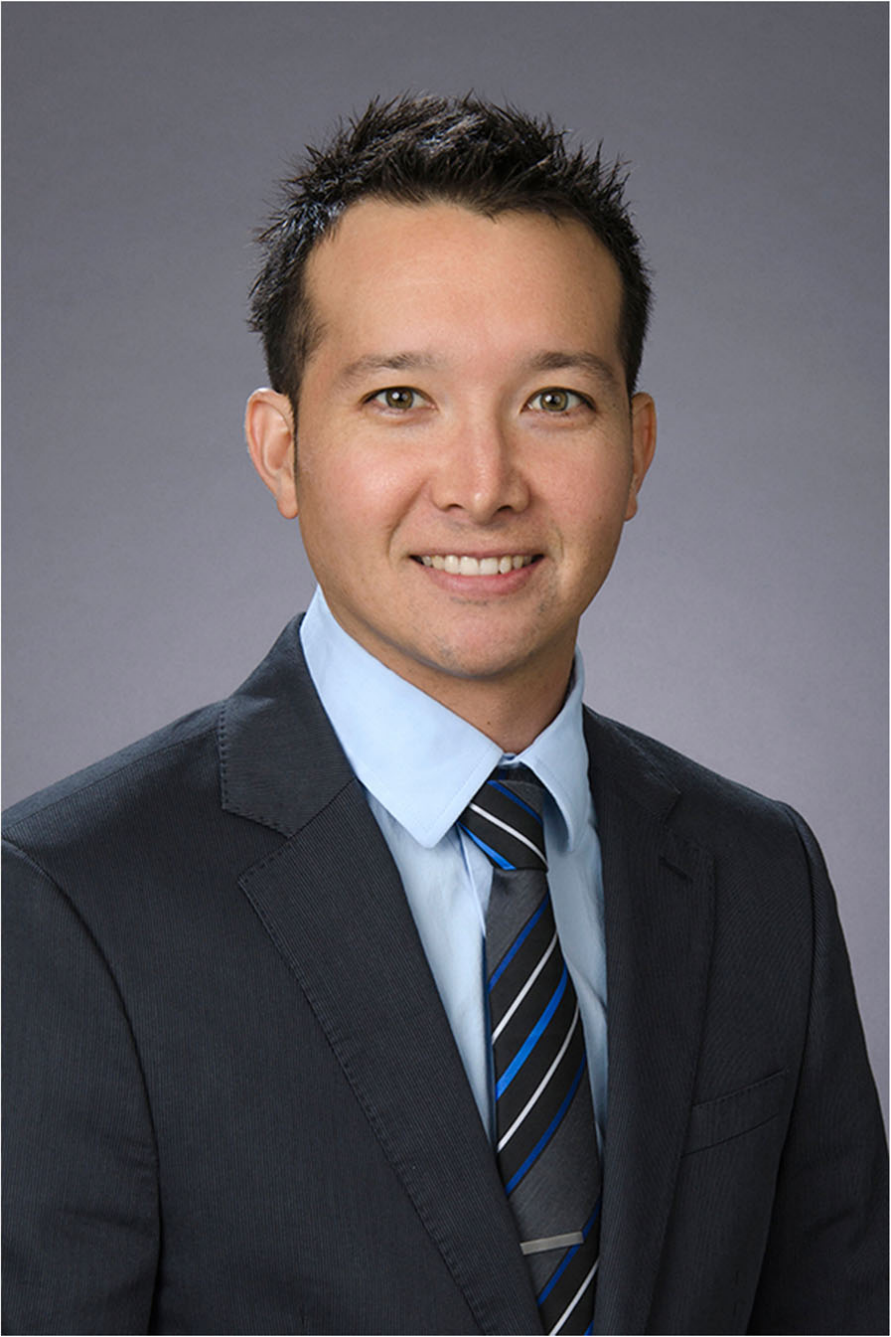
Tim K. Mackey is the Director of the Global Health Policy Institute, an Assistant Professor at UC San Diego – School of Medicine, and the Associate Director of the Joint Masters Degree Program in Health Policy and Law at UCSD-California Western School of Law. He has a multidisciplinary background and his research focuses on global health policy, law, governance, and diplomacy and has worked or consulted for organizations including the World Health Organization, U.S. Department of State, and the U.S. Department of Justice

In 1996, former World Bank President James Wolfensohn made a groundbreaking speech calling for international action and attention to deal with what he coined the ‘*cancer of corruption*’ [1]. Decades later, this representation of corruption as a destructive disease seems fitting, as health-related corruption is now a multifaceted, multijurisdictional, and multibillion dollar phenomenon that threatens the future progress of global health [2, 3].

Similar to cancer, health-related corruption comes in several types (ranging from “petty” corruption such as absenteeism of healthcare workers to “systematic” corruption involving multinational companies engaged in widespread healthcare fraud and abuse, and “grand” corruption occurring at high levels of government), can invade and spread (infiltrating public and private sectors as well as poorer and richer countries alike), has an enormous financial cost, is often difficult to detect/diagnose and, most importantly, is hard to treat [2, 3]. Critically, health-related corruption is distinctly dangerous compared to other forms of corruption in traditional economic sectors such as energy, extractive industries, banking, and construction, in that it presents a “dual-burden” of limiting both economic/human development while at the same time endangering patients and population-level health [2, 4].

**Fig. 2:**
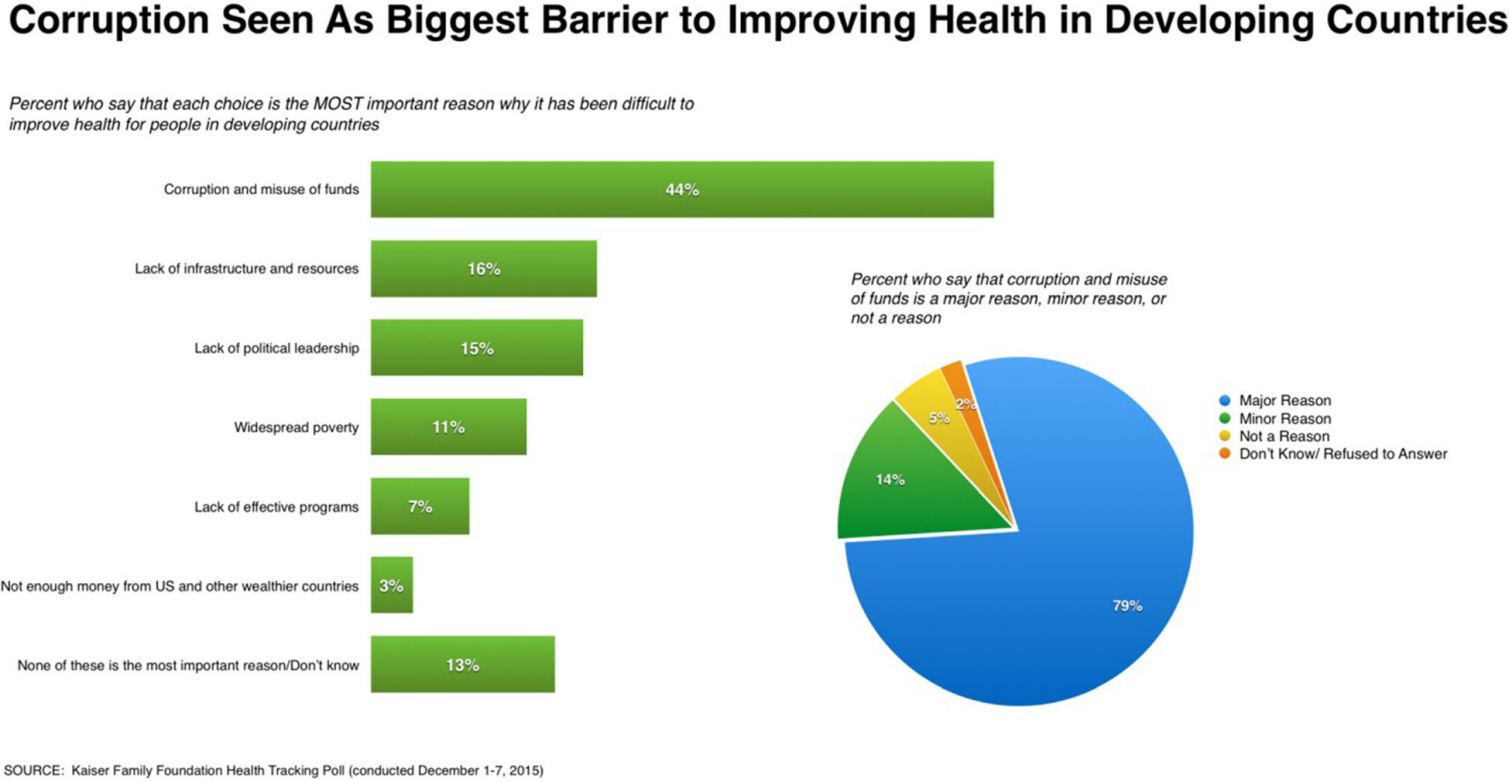
Public perception on the role of corruption in improving health in developing countries (Kaiser Family Foundation) [6]. Surveys conducted by the Kaiser Family Foundation examining Americans’ opinions on the US role in global health have consistently found that the American public views corruption as a major problem. In its 2015 survey, 44 % of respondents believed that ‘*corruption and misuse of funds*’ was the most important reason why health cannot be improved in developing countries. Seventy-nine percent of respondents also believed corruption was a major barrier, meaning that corruption is viewed by the American public as the biggest barrier (more than lack of infrastructure/resources, poverty, lack of political leadership and effective programs, and lack of funding) to investing in programs that support global health goals

**Fig. 3:**
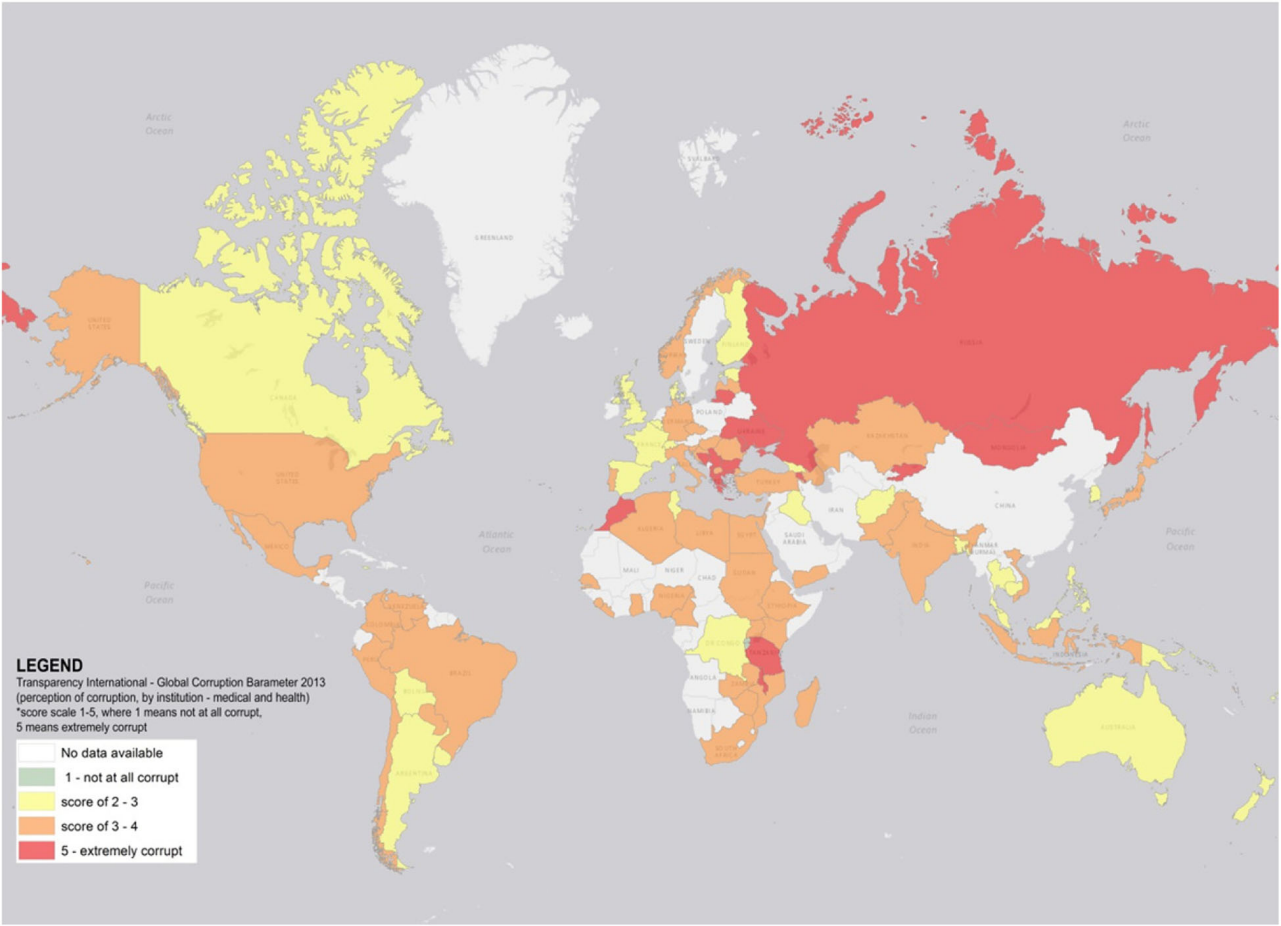
Heat map of Transparency International’s Global Corruption Barometer (GCB): perceptions of the extent of corruption in medical and health services institutions. Transparency International’s 2013 GCB uses surveys from more than 114,000 respondents in 107 different countries to assess people’s direct experiences and views on corruption in main institutions in their countries. This includes assessing perception of the extent of corruption in Medical and Health Services institutions measured on a scale of 1 to 5, where 1 indicates “not at all corrupt” and 5 indicates “extremely corrupt.” The above map was generated using publicly available data from GCB and was visualized in ArcGIS map. It depicts the varying levels of public perception on how corrupt medical and health institutions are within respective countries (global mean score of 3.3)

The cost of health-related corruption can extend beyond the people and communities it directly impacts, as the mere presence of corruption can lead to negative public perception and criticism about the role of foreign health aid [5]. This is evidenced by surveys conducted by the Kaiser Family Foundation that have consistently found that corruption and misuse of funds are seen as the largest barrier to improving health in developing countries among the US public (Fig. 2) [6]. Transparency International (TI), an international non-governmental organization created to combat corruption, has also explored perceptions of corruption in different public institutions, including in the medical and health sector. Results from its 2013 Global Corruption Barometer (GCB) [7] indicate that perceptions of the extent to which the medical and health services sectors are affected by corruption vary widely across different countries (Fig. 3). Collectively, these negative views can unjustifiably inflate public concerns about the effectiveness of development assistance for health, leading to lowered government commitment to health aid for developing countries that depend on these humanitarian investments [5].

The motivation of different actors, including government officials, private companies, and organized crime groups to engage in health-related corruption should come as no surprise: the healthcare sector is one of the fastest and largest segments of the global economy, accounting for nearly 10 % of the worldwide gross domestic product (GDP) according to the World Bank [8]. In addition, the health sector is characterized by unique risk factors and inherent complexities particularly susceptible to corruption, including information asymmetry, the large number of actors and mix of public and private sectors in healthcare systems, market uncertainty, and large amounts of public spending [2–4]. These vulnerabilities allow the presence of various types of corruption, spanning from bribery, kickbacks, and informal payments to health personnel/administrators; fraud and abuse involving payments for healthcare goods and services that are not rendered; collusion and bid rigging in healthcare procurement and contract awards; biased or unfavorable decisions due to conflicts of interest in healthcare transactions/relationships; corruption in medical practice, education, and research; and diversion, embezzlement and theft of various healthcare resources [2–4, 9–12]. Further, the diversity and scope of health-related corruption makes it equally difficult to design programs effective in preventing, detecting, and controlling corrupt practices [2].

The challenges of health-related corruption are further accentuated in the context of global health programs and settings. Specifically, global health programs are transnational in nature, including participation of one or more countries, and often involve substantial foreign aid and multiple development partners. Additionally, many global health programs operate in countries with weak governance or rule of law [2, 13]. These factors can lead to greater vulnerabilities for infiltration of corruption that is multijurisdictional, impacted differently by the varying policies, laws and regulations, and influenced by local social and cultural beliefs about what constitutes corrupt acts [2, 9]. There is also a great deal of money at stake, with development assistance for health experiencing a rapid increase from a mere US$ 11 billion in 1999 to the US$ 36 billion disbursed in 2015, marking the emergence of global health as a multibillion dollar sector [14].

In an attempt to raise awareness to the unique challenges of global health corruption, this Forum article presents views from a set of multidisciplinary experts from fields including public health, political science, economics, and international development. Our contributors comprise a mix of practitioners, implementers, and researchers from civil society and global health institutions, with experience working for organizations directly engaged in anti-corruption programs such as the World Bank, TI, and the UN Development Programme (UNDP). The aim of this Forum is to bring together these different perspectives to identify key priority areas that urgently need attention and to lay out a roadmap for global health anti-corruption efforts in the post-2015 development agenda.

The following key themes relating to how to advance anti-corruption goals emerged from our discussions:

1. **Problems with the concept of “zero” corruption:** Corruption is endemic in all health systems, including rich and poorer countries. However, anti-corruption initiatives that aim for “zero” tolerance of corruption may penalize programs that are putting in place the building blocks for more effective and corruption-resistant health systems. Harsh penalties may create perverse incentives to hide corruption, rather than rooting it out.
2. **Better data:** A pervasive theme among all contributors was the admission that the true scope and cost of global health corruption is largely unknown. Corruption can be invisible, difficult to detect, and often highly politicized, all of which require better indicators, data collection/reporting, and analysis.
3. **Importance of transparency:** Transparency is a critical tool in curbing health corruption. This includes enhancing transparency and disclosure in financial systems and controls, healthcare relationships/transactions, and health sector procurement systems.
4. **Multi-stakeholder partnership:** Many actors, including governments, private sector, and civil society, have an interest in controlling corruption. Thus, multi-stakeholder partnerships hold promise as a strategy for advancing transparency and accountability. Coalitions of local, national, regional, and international stakeholders in both the public and private sectors (including civil society) may help to increase trust and gain the political support needed to ensure that healthcare services and projects are protected from corrupt practices.
5. **Linkage to global health security:** Combating global health corruption is paramount to international investments and shared goals to secure national and global health security arising from the threat of infectious disease outbreaks (such as the recent Ebola outbreak) and other health emergencies.
6. **Governance is important:** “Good” governance must encompass anti-corruption efforts, including governance at the national level, governance of global pharmaceutical supply chains, and governance of the international development agenda. This is particularly true with the new United Nation’s Sustainable Development Goals (SDGs), which, for the first time, specifically address the themes of corruption, ensuring access to healthcare services and medicines, and encouraging global multi-stakeholder partnerships as key strategic goals.

International attention concerning corruption has been steadily growing, including a recent 2015 anti-corruption summit hosted by former UK Prime Minister David Cameron. Yet, insufficient attention has been focused on the health sector and particularly on global health, despite the fact that global health corruption represents a significant barrier to the achievement of universal goals of promoting human health, economic development, security, and poverty alleviation.

In response, it is critical that the international community develop a unified framework devoted to combating global health corruption as the disease that it is. These efforts should be underpinned by SDG 3 (“Ensure healthy lives and promote well-being for all at all ages”, SDG 16 (sub-target 16.5, “Substantially reduce corruption and bribery in all their forms”), and mobilized through robust global multi-stakeholder partnerships as encouraged under SDG 17 (“Strengthen the means of implementation and revitalize the global partnership for sustainable development”). Global partnership should look to leverage all anti-corruption resources, programs, tools, law/policies, and initiatives the international community has at its disposal.

Global efforts to address global health corruption could be operationalized under a newly formed United Nations High-level Panel on Corruption, convened by the Secretary General, that would include in its programmatic objectives a specific review of the impact of global health corruption on human health, human rights, security, and international development. The panel should include partnership with key institutions that have been active in the fight against health corruption. The proposed panel should deliver a set of recommendations for concrete solutions, development of SDG indicators that specifically measure health-related corruption, encourage anti-corruption policy coherence, and establish a roadmap for achieving health systems that are liberated from the chains of corruption.

## Foreign aid, global health programs, and corruption

### William D. Savedoff (Fig. 4)

**Fig. 4:**
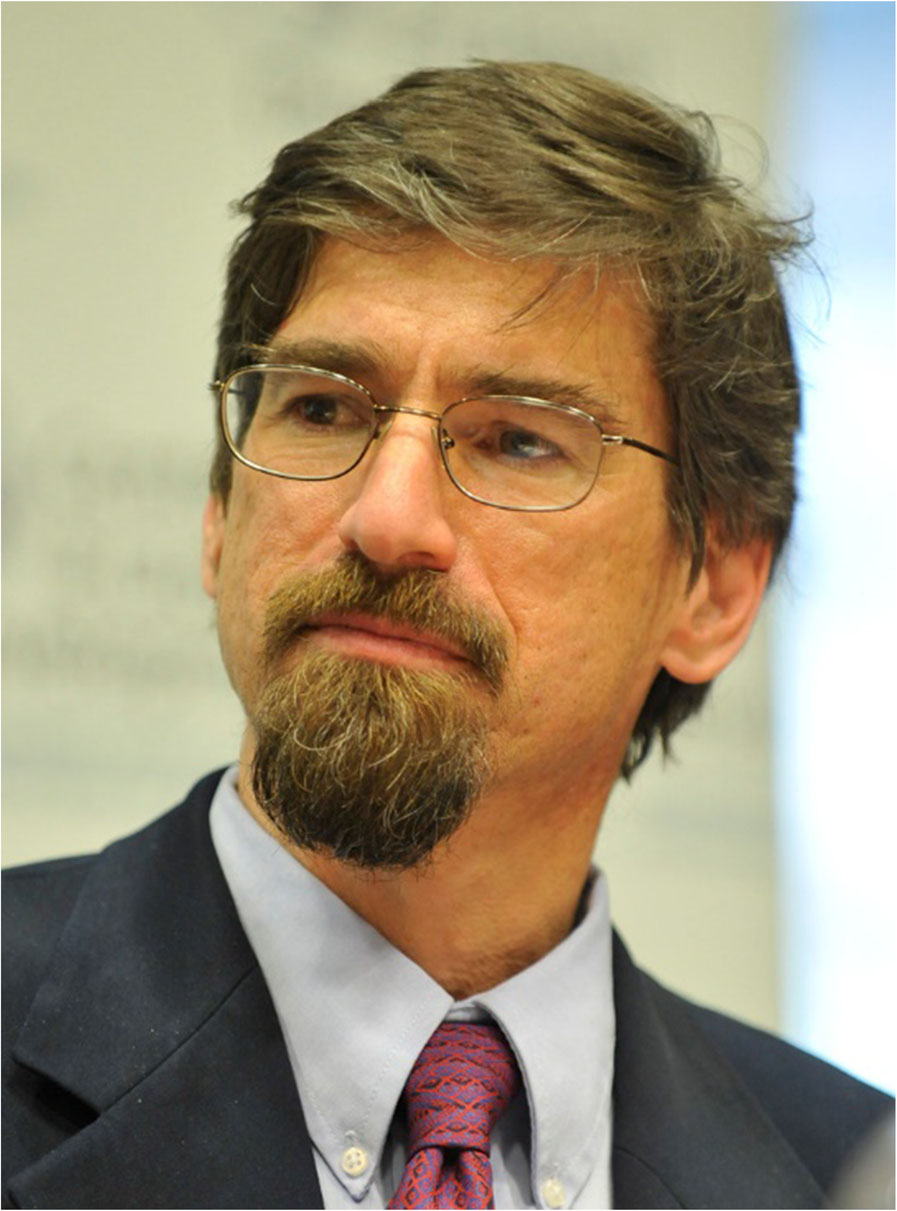
Bill Savedoff is a senior fellow at the Center for Global Development (CGD), where he works on issues of health policy, performance payments, and corruption. Before joining CGD, he worked at the Inter-American Development Bank and the World Health Organization on projects and research in Latin America, Africa and Asia. His publications include *The Health Financing Transition*, *Governing Mandatory Health Insurance*, and *Diagnosis Corruption*

Corruption is a problem for health programs worldwide, yet we know surprisingly little about its scale and impact. Without this information, we do not know whether anti-corruption strategies are doing too much or too little, whether they are effective or weak, or whether they improve program impact or get in the way.

Worldwide, foreign aid programs have been remarkably successful in improving health conditions, even in extremely corrupt settings. Foreign aid has been essential to the eradication of smallpox, prevention of vaccine-preventable diseases like measles, treatment of potentially lethal conditions like diarrhea, and expanded access to services that improve maternal and infant health [15, 16]. This kind of success resonates with taxpayers in wealthy countries who strongly support aid for health programs; nevertheless, they worry about corruption. For example, 60 % of Americans think US global health spending is “too little” or “just right”, but 44 % believe “corruption and misuse of funds” to be the most important reason behind health aid ineffectiveness (Fig. 2) [6].

Corruption certainly affects health aid, but it also affects all health systems to some degree [3]. In richer countries, corruption tends to make healthcare delivery costlier, while in poorer countries, it tends to undermine the delivery of care and exacerbate inequities. In low- and middle-income countries, petty bribes and absenteeism are well documented, as are occasional cases of high-level embezzlement and kickbacks. Experience shows that foreign aid cannot solve these problems of corruption without political commitment from the receiving countries [17, 18], but it can improve healthcare delivery and population health even in very corrupt contexts [19].

The primary approach used by donors to assure integrity in their operations is to control how aid funds are spent and monitored. Usually, recipients must establish separate accounts, reporting systems, and bidding procedures. Recipients may even have to obtain prior approval from donors before issuing requests for proposals. This has a positive side: following such procedures can improve local capacity to receive, manage, and spend funds appropriately. Nevertheless, financial controls can also raise costs and encumber implementation. In 2010, more than 90 % of USAID contracts went to US-based consulting firms, in part because these firms could manage the agency’s complex bidding and reporting requirements. At the World Bank, one study found that contracting consultants took 17 months for programs that only lasted about 2 years [20].

Aid agencies do need procedures to ensure integrity but current approaches are unbalanced because they aim for “zero” corruption without regard for results, namely the impact on healthcare delivery and population health. For example, Germany, Spain and Denmark suspended contributions to the Global Fund to Fight AIDS, Tuberculosis and Malaria in 2011 after a media report exaggerated the scale of corruption detected by the Fund’s own inspector general’s office. To show they were tough on corruption, donors halted funding without regard to the severity or impact of their actions on program results. In doing so, they also penalized the Global Fund for its efforts at integrity and transparency [5]. In their zeal to root out corruption, investigators can also lose sight of what health programs are trying to accomplish. In 2013, a report from the Special Inspector General for Afghanistan Reconstruction called for USAID to suspend a very successful health program because they found inadequate accounting systems within the Afghan Ministry of Health. The report not only lacked specific evidence of fraud; it also failed to consider how a program at risk for corruption could have contributed so much to increases in healthcare delivery and reductions in child mortality [5].

Ignoring information about program results when fighting corruption endangers progress. Simultaneously, it neglects a powerful tool for detecting fraud and improving anti-corruption strategies. If agencies did a better job of measuring results, they could use this information to prioritize how they allocate anti-corruption resources. They could also use such information to learn how anti-corruption strategies affect project success so as to make them more effective and less intrusive. Finally, results measurements can help aid agencies to distance themselves from subjective and arbitrary judgments about the trustworthiness of partner governments and about suspending aid.

Global health programs are well worth the money. The world should invest more in expanding access to healthcare, disease prevention, and global public goods like epidemiological surveillance and advance preparation for outbreaks of epidemics like SARS, highly pathogenic influenza, Ebola, and Zika. Fortunately, global health programs succeed despite corruption in many contexts. Aid should continue to support health programs but with greater attention to measuring results as a way to highlight when corruption is an obstacle and to acknowledge when it is not.

## Economics, health systems, and corruption

### Maureen Lewis (Fig. 5)

**Fig. 5:**
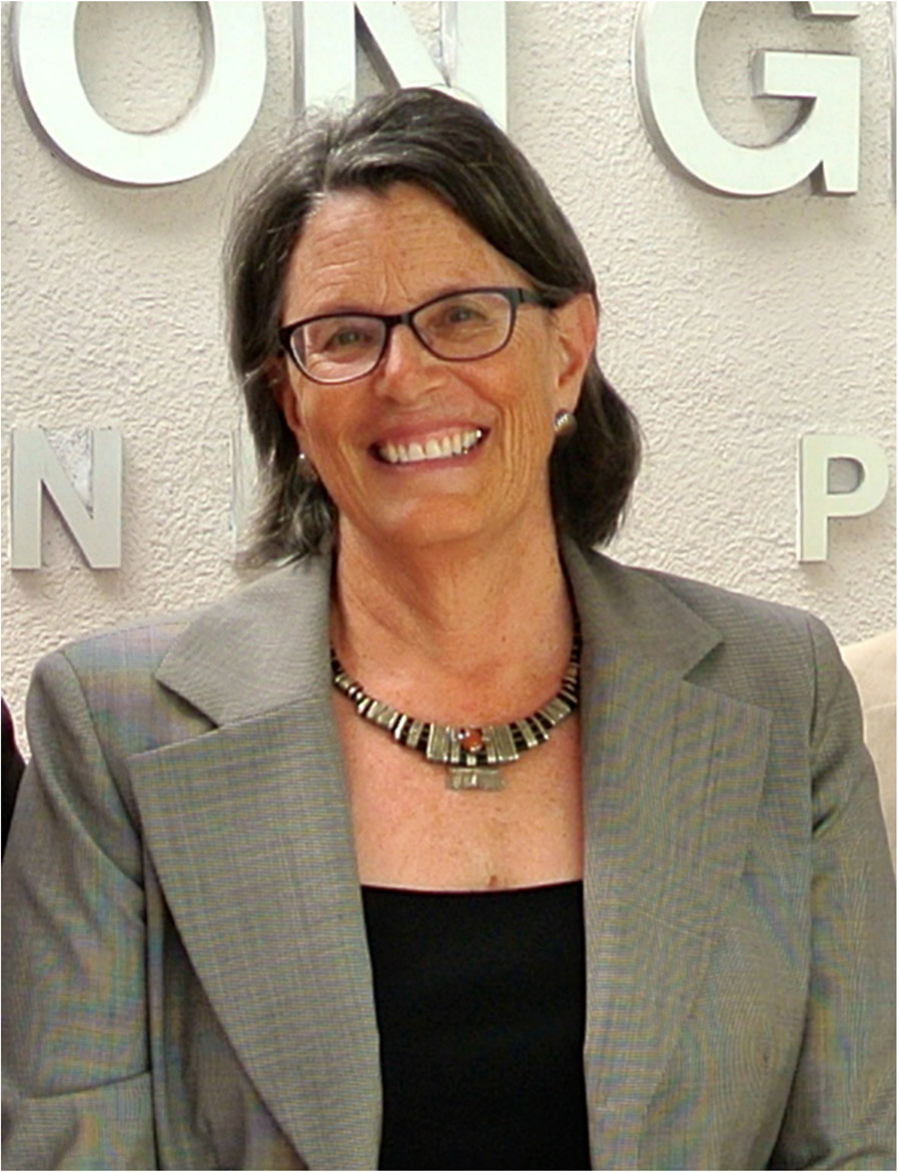
Maureen Lewis is the co-founder and CEO of Aceso Global, a non-profit organization that strengthens health systems in emerging markets and developing countries by improving hospital management and integrated care, quality, and performance. She is a non-resident Fellow at the Center for Global Development and a Visiting Professor at Georgetown University’s School of Foreign Service. Prior to that, Dr. Lewis spent 22 years at the World Bank in management and staff positions, most notably as Chief Economist Human Development. Dr. Lewis was a Senior Fellow at the Center for Global Development and was previously a Senior Research Associate at The Urban Institute working in Latin America

Healthcare systems underpin both healthcare delivery and efforts towards attaining universal healthcare (UHC), the global goal for public health organizations such as the World Health Organization (WHO). Any push to attain UHC can founder on shifting sand. Infectious diseases like malaria and HIV dominate the donor and private foundation landscape in developing countries, but chronic conditions, including cancer, cardiovascular disease, diabetes and accidents, are eclipsing communicable diseases as causes of morbidity and mortality across the globe. On the one hand, this shift represents a remarkable achievement in controlling infectious diseases, on the other, prevention and treatment of chronic diseases imply management of more complex morbidities and more complicated services.

The performance of healthcare systems determines the effectiveness and costs of healthcare services. Corruption is a significant cost driver and a cancer in undermining effective healthcare services. The Ebola outbreak, for example, stemmed from weakened public health systems suffering from decades of weak institutions and conflict making conditions susceptible to corruption and mistrust [21]. As demonstrated in heavily impacted countries of Liberia and Sierra Leone, failures in patient diagnosis and treatment can reflect problems in health system functioning, specifically its clinical, non-clinical, and management tasks. Economists worry about the costs and effectiveness of services – is there too much or too little care being provided, are services organized and delivered efficiently, are resources used most effectively to meet needs, and is performance where it should be? Effective health systems explicitly and implicitly intend to address many of these concerns because they bolster access and performance of clinical services.

Over the past two decades, the honesty and integrity of healthcare systems across low- and middle-income countries has troubled citizens, external and internal observers, and governments alike. Coming from a broader agenda of corruption and development that linked poor services and slow growth to widespread corruption [22], the health sector has had to confront corruption in healthcare systems. Initially, researchers and policymakers implicitly assumed that corruption was not a problem in the health sector, and organizations like the World Bank determined that investments in health and education were the preferred options in corrupt societies as they implicitly believed these sectors were immune. That assumption no longer holds and evidence bears this out.

Corruption can be defined in abbreviated terms as ‘*use of public office for private gain*’ [23]. However, what has led to corruption in healthcare? Fundamentally, a lack of accountability. This lack of accountability derives from a number of factors, including inadequate management, lack of oversight, poor training, and an absence of performance incentives, which in turn make accountability impossible [24]. Accountability is fundamental as it requires that “*officials are called to account and to answer for responsibilities and conduct*” [25], that is, it ensures consequences for poor behavior and ideally rewards exceptional behavior. Because accountability in most healthcare systems is diffused across patients, payers, managers, and citizens, there is effectively little if any accountability to anyone. Without accountability, public servants face few restraints. Common measures of corruption in healthcare across low- and middle-income countries include absenteeism of physicians and nurses (a practice rife in much of the world), health workers, including physicians, forced to purchase their public sector jobs, ghost workers, frequent “stock outs” of drugs and supplies, leakages of public monies, patients paying “under the table” directly to individual providers, and a perception of healthcare as among the most corrupt sectors in many countries [9, 24]. Such practices and circumstances compromise the delivery of healthcare.

The leap to how corruption undermines healthcare systems should be obvious. Without personnel, drugs, management, and other inputs, healthcare services are effectively unavailable. For economists, this scenario translates into total system breakdown because resources are being wasted, performance is poor, outputs are compromised, and expected outcomes remain well out of reach. Indeed, corruption introduces serious complications as it undermines every aspect of healthcare delivery from the effectiveness of providers to the availability of inputs for the care of patients [3]. A move to address any breakdown in healthcare entails efforts on multiple fronts.

Numerous public initiatives have attempted to mitigate the observed consequences of corruption. A sampling of these include reducing costs by bulk purchasing of supplies and drugs, and public hiring and management of personnel in order to keep human resources “in-house” [24]. These initiatives reflect efforts to internally manage and control healthcare delivery to safeguard basic standards and improve quality. However, these efforts may have had the opposite effect. They have served to fuel corruption and erode quality precisely because institutions, managers, and employees are not held accountable by the public healthcare system.

Absent from much of the healthcare agenda is an acknowledgment of any perverse implicit or explicit incentives that allow for poor behavior. Economists rely on incentives to encourage good performance through, for example, merit promotions or bonuses for good performance, or to discourage unethical or illegal behavior such as stealing of drugs, absenteeism or financial mismanagement through sanctions, demotions or firing. However, these incentives remain rare in public systems even when egregious performance is documented. Despite the common absence of incentives, well-designed explicit incentives with clear accountabilities remain fundamental to well performing healthcare systems. Evidence increasingly points to separating the payer and provider to allow oversight by a different entity, and to contracting out services spanning clinical care to facility maintenance to private or publicly accountable entities [24].

Healthcare is among the most complex sectors in any economy. Raising the bar and improving how these systems work will hinge on clear incentives and effective accountability that roots out the various forms of corruption that have infiltrated the health system of this trillion-dollar global sector. Without that synergy, clinicians, citizens, and economists will never be satisfied, nor should they be, with healthcare locally and globally.

## Civil society fights corruption in healthcare

### Frank Vogl (Fig. 6)

**Fig. 6:**
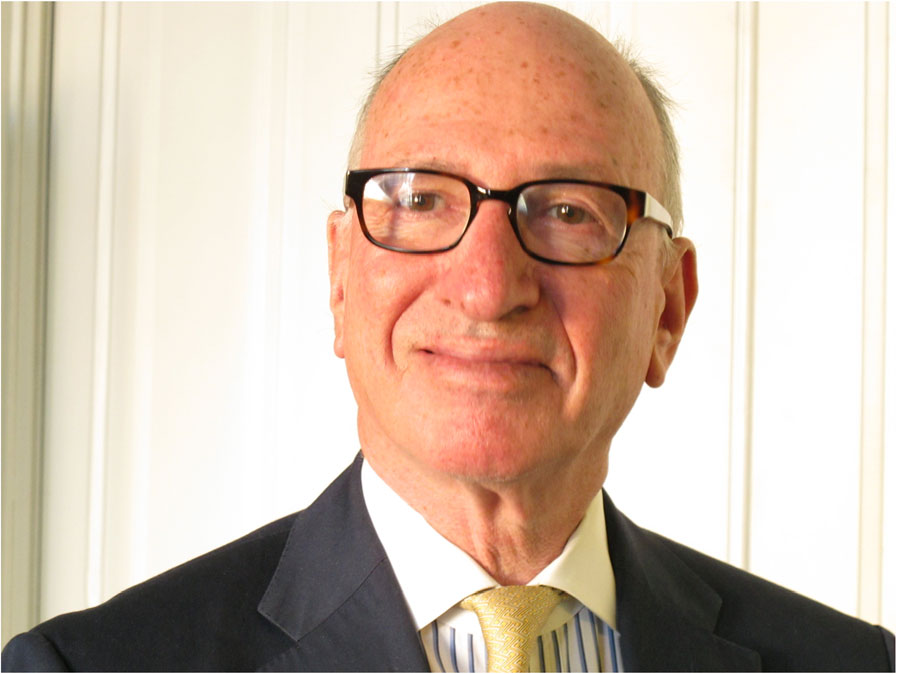
Frank Vogl is a co-founder of Transparency International and the Partnership for Transparency Fund and serves as an advisor to both organizations. He is an adjunct professor of government at Georgetown University. Frank is the author of *Waging War on Corruption – Inside The Movement Fighting The Abuse of Power* (new paperback edition, September 2016) updated, 2016) Rowman & Littlefield. He writes and lectures extensively on corruption – www.frankvogl.com

Concerns about the failure of a large number of well-intentioned official foreign aid programs and projects in the healthcare sector were one of the powerful drivers behind the establishment of TI in 1993. TI was the first global non-governmental organization dedicated exclusively to anti-corruption, and it currently operates through national chapters in more than 100 countries.

Today, many civil society organizations are planning and implementing anti-corruption projects to specifically improve healthcare services, notably for the poor in poor countries. The scale of the challenge is enormous; for example, TI’s 2016 survey for nine countries in the Middle East and North Africa showed that 20 % of citizens paid bribes to receive health services, with the rate at 38 % in Morocco [26]. The GCB for sub-Saharan Africa found that 12 % of citizens routinely paid bribes for health services, and in many cases they paid multiple bribes, notably when needing hospital services [27].

An important challenge is to find ways to obtain first-hand reports from citizens on the corruption that they encounter in healthcare services and to bring this to the attention of public officials. Over the last couple of years, the Partnership for Transparency Fund (PTF), an independent organization originally started in 2000 by the founders of TI, has been pioneering a new information and communications technology (ICT) approach in Uganda. Its likely success can lead to similar projects in other countries. Namely, PTF, together with the Anti-Corruption Coalition Uganda, launched the Citizen Action Platform (CAP) [28] to deploy ICT to systematically record, aggregate, map, and track cases of corruption through to their resolution. The aim has been to provide citizens with a means to safely and anonymously report abuse from their mobile phone and receive feedback. The ICT approach has dramatically reduced the costs of monitoring and reporting public service failures, which provides civil society organizations with sufficient solid data to constructively engage with service providers through a better understanding of where, when, and what issues citizens are most concerned about. The CAP program gained traction after instituting a partnership with UNICEF’s Ureport program in January 2016, and may serve as a model in developing more accountable and transparent means of providing healthcare services and distributing medicine and medical supplies. While the reports received often relate to waste and inefficiency in services, more than 25 % of all complaints under the CAP program included bribe taking.

PTF has been involved in engaging citizens against corruption on many fronts in more than 50 countries through specific projects. Experience from PTF projects in the health sector where, in many cases, demands for bribes by officials and healthcare workers undermined service delivery has yielded valuable lessons. PTF has shared these findings widely [29, 30] and they have, for example, influenced some of its most recent work, such as the CAP program. Accordingly, PTF has found, for example, that key approaches in implementing citizen-led projects in the health sector where waste of resources, inefficiency and corruption are commonplace, include:

- Raising public awareness of rights, particularly the costs of medicines and treatments, is a key first-step to ensuring these rights are appropriately fulfilled.
- Designing projects to cover a wide range of issues so that they are capable of hearing a wide variety of citizen voices and responding to their greatest concerns – this proved to be most effective, for example, in PTF’s work with 15 communities in service delivery projects in India.
- Engaging constructively with authorities is the most effective way to resolve issues and achieve change.
- Advocacy is more powerful with partnerships between civil society organizations at the national level, who have access to decision-makers, and the local level, who can ensure that service delivery is supported by systemic or policy changes.
- Trained and supported volunteer citizen committees can be powerful agents to identify corruption and push for improvements, even on technical issues.
- Anti-corruption commissions and public service codes of conduct can be helpful in elevating corruption issues and strengthening accountability among service providers.

Tragically, progress in improving healthcare delivery in many countries suffers not only from the corruption that PTF and its partners have been addressing community-by-community, but also because of grand corruption – the wholesale theft of health budgets by senior government officials and politicians. At the level of grand corruption there is no meaningful way to single out the impact on healthcare relative to overall economic development and the provision of basic services to all citizens to reduce poverty. The scale of this problem is well highlighted by the African Progress Panel Report 2013 [31], which concluded that grand corruption was the prime cause of the extraordinary poverty in many of the natural resource-rich countries of sub-Saharan Africa – core health data for Nigeria and Angola, for example, are atrocious, especially when the oil wealth of these countries is considered.

For TI, the specific efforts made by many of its national chapters to implement healthcare projects, plus the thousands of complaints they seek to handle from individual citizens who bring forward personal cases of corruption, go hand-in-hand with a global “No Impunity” strategy. We believe that far more effort must be made by the international community to ensure that top government officials and politicians, as well as the business people they conspire with, no longer operate as if they are above the law.

## Emerging tools and health system interventions to prevent corruption – a role for open contracting

### James Sale (Fig. 7)

**Fig. 7:**
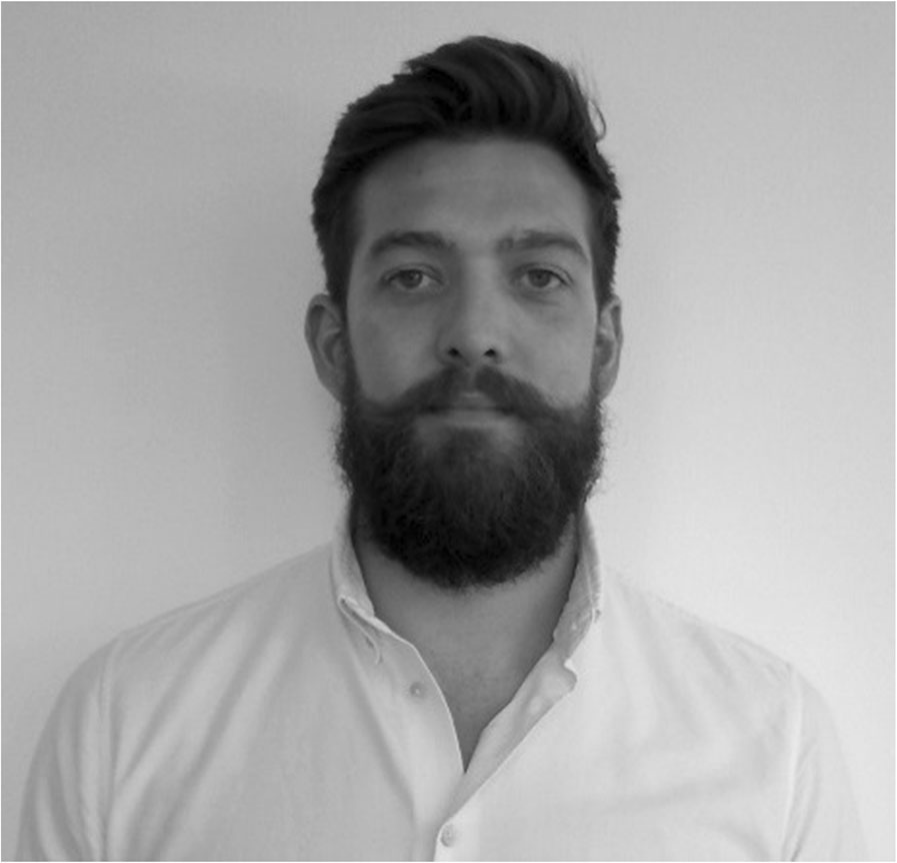
James Sale is the Program Manager for Transparency International’s Pharmaceuticals & Healthcare Program. He joined TI in 2014 to establish the Pharmaceuticals & Healthcare Program having previously worked in governance and public financial management at Crown Agents and vaccine surveillance with the World Health Organization

Of the trillions of dollars spent on healthcare globally on an annual basis [32], a large proportion is spent through large public contracting for medicines, equipment, and health facility construction. However, estimates suggest that 10–25 % of global spending on public procurement is lost to corruption and waste [3]. It is therefore germane to look at procurement when considering emerging health sector-wide anti-corruption tools.

Health sector procurement is particularly vulnerable to corruption due to its technical complexity, numerous stages, and requirement of high expertise. It is universally accepted that a fundamental practice for curbing corruption in public procurement is increasing transparency. This is nothing new; however, what is new is the growing use of open contracting as a pragmatic remedy to a lack of transparency as part of the wider move towards open governments. Open contracting is the practice of publishing and using open and accessible information from key stages of the procurement process. In health systems, this can begin with publishing needs assessments and continue through to quality assurance and contract completion [33]. This information is only useful if easily applied to identify potential issues and hold procurement agents accountable. To achieve this, data needs to be publically accessible according to measures such as the Open Contracting Data Standards, so that external oversight can be properly carried out [34].

At the 2016 Anti-Corruption Summit in London, open contracting in public procurement gained substantial support with a commitment in the Summit Communique to ‘*making public procurement open by default – so that citizens and businesses can have a clear public record of how public money is spent*’ [35]. Furthermore, four countries (Argentina, Malta, Mexico, and Nigeria), supported by a UN commitment to work with ‘*global, regional and country initiatives that strengthen the transparent procurement of health commodities*’ , committed to progressing open contracting standards in their health sectors [36]. These pioneering countries are backed by a genuine appetite for reforming health sector procurement in many more countries. To encourage more to follow this lead, the added benefits of reducing procurement corruption through increased transparency need to be demonstrated. For example, disclosing adequate levels of data and information can produce greater purchasing power for governments through the knowledge of what others are paying, allowing them to achieve better value for money and reducing the risk of price gouging, price manipulation, and overpayments [4].

In 2014, hundreds of thousands of Ukrainians died due to a lack of essential life-sustaining medicines, affecting those suffering from tuberculosis, viral hepatitis, hemophilia, and orphan diseases [37]. One major contributing factor was the failure of the public medicines procurement system, which the Ukrainian government itself called ‘*inefficient, corrupted, non-transparent*’ [38]. In response, the Ukrainian Ministry of Health outsourced the procurement of drugs to two UN agencies (UNDP and UNICEF) and Crown Agents, a British social enterprise working in international development. The organizations reformed the system to meet international standards and have already reported large savings and increased flow of medicines to patients. UNDP reported US$ 1 million of savings in anti-tuberculosis medicines this year alone, and Crown Agents were able to procure oncology medicines at prices 45 % cheaper than the Ministry of Health paid in 2014, saving nearly US$ 20 million [39, 40].

In addition to the basic reform of medicines procurement, Ukraine has successfully launched the e-procurement platform ProZorro [41]. Formed by a public-private partnership including TI Ukraine, the system is based on the Open Contracting Data Standards and has won international awards. Already having processed some health sector contracts and demonstrated savings, ProZorro will be mandatory for all public procurement as of August 2016. As with any new system, there will undoubtedly be improvements that need to be made; however, it is an extraordinary accomplishment to create such a system in the context of political and security instability. This sets a precedent for others.

While national governments are seen as the key drivers for improving procurement systems, those acting at a regional and global level are equally crucial for progress. These key actors need to not only lead by example, but must also have the resources to invest in innovative solutions and wider adoption. One such innovative approach is currently being launched by the Global Fund to fight AIDS, Tuberculosis and Malaria (The Global Fund).

Wambo.org is an e-procurement platform that acts as an e-marketplace for Principle Recipients of Global Fund grants to purchase quality-assured goods launched in 2016 [42]. The system pools orders and, by combining the purchasing power of governments, aims to keep costs low and consistent. Wambo.org is also set to roll out beyond just Global Fund grantees, including non-for-profit organizations, with The Global Fund projecting savings of at least US$ 250 million over the next 4 years. Wambo.org is an online procurement system that provides information on products, prices, delivery times, and tracking [43], much like an online shop. While principally acting as an e-marketplace, systems such as Wambo.org can also record the type of data that is needed for external oversight and accountability. When adequate public procurement data is disclosed in a usable format, civil society is able to scrutinize and identify corruption risks. Data collected through such e-procurement processes should be publically disclosed and accessible for further study.

Despite these types of examples, current anti-corruption tools and interventions are still limited, and there is an absence of key actors committed to preventing corruption from occurring in health systems. Corruption remains rife and immediate action is required in order to coordinate a holistic and multi-stakeholder approach. Until such action, progressive tools will have little impact and success will occur in isolation.

## Why making the invisible visible matters for global access to medicines

### Jillian Clare Kohler (Fig. 8)

**Fig. 8:**
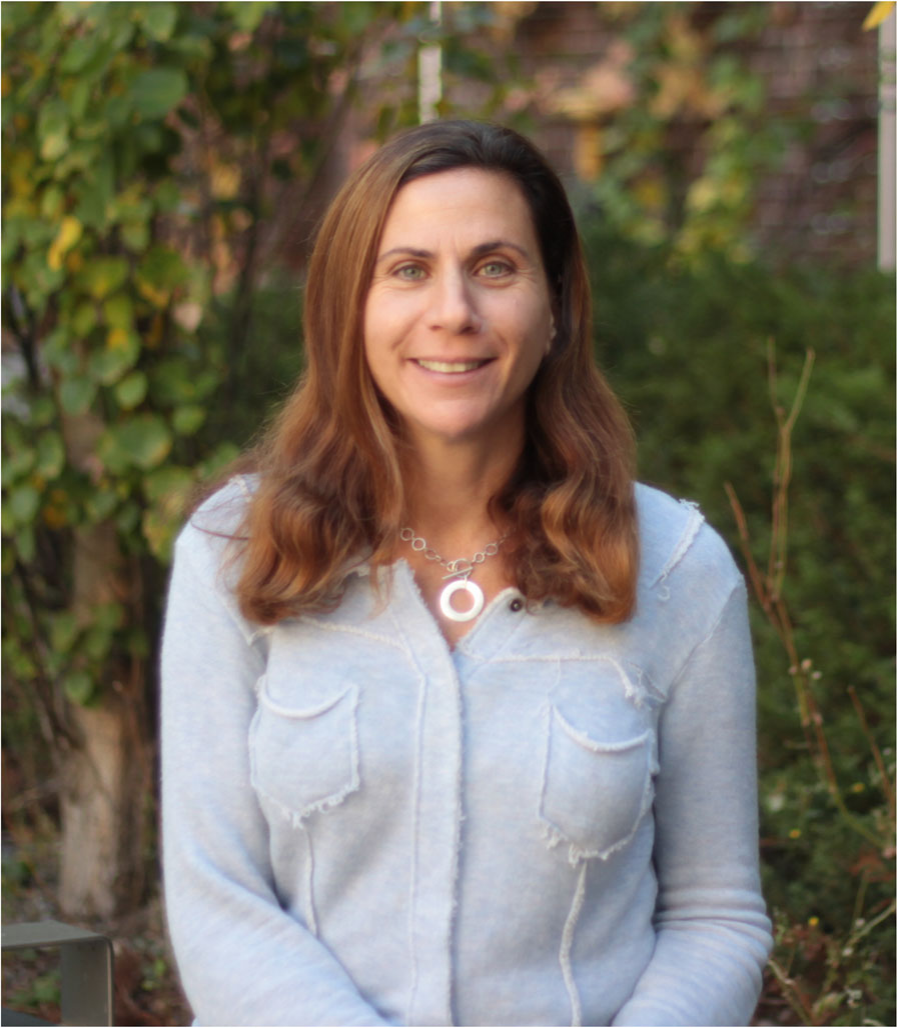
Jillian Clare Kohler is a Professor at the Leslie Dan Faculty of Pharmacy, the Dalla Lana School of Public Health and the Munk School of Global Affairs. She is also Director of the WHO Collaborating Centre for Governance, Transparency and Accountability in the Pharmaceutical Sector. Her research and teaching are focused on global pharmaceutical policies related to improving fair access of those in need to critical medicines. Prior to joining the University of Toronto, she worked exclusively on global pharmaceutical policy for a number of UN organizations, including UNICEF, the World Bank and the WHO. She continues to advise global institutions and NGOs on global pharmaceutical policy issues such as anti-corruption strategies, drug regulations, and reimbursement policies

Uneven access to pharmaceuticals continues to be a serious global health challenge despite targeted investments by the development community in programming and services. As one illuminating example, 22 million people living with HIV remain without access to antiretroviral therapy despite rapid scale-up and increased availability of generic products [44]. We know that improved access to medicines (and vaccines) could save as many as 10 million lives per year [45]. Why then do we have persistent disparities in access to medicines? Much of the development policy conversation on, and interventions designed to address, medicine barriers have focused traditionally on infrastructural limits to service delivery and the impact of intellectual property; yet, there is an increasing body of evidence that illuminates how governance challenges may create opportunities for corruption and result in additional barriers to access to medicines [46, 47].

Further complicating issues is the inherent complexity of the pharmaceutical system, which encompasses the actions of public and private stakeholders as they move drugs through the global supply chain from purchasing to delivery to patients. The system is inherently challenging to govern, as it is characterized by multiple opportunities for system failure, limited accountability between stakeholders, and a lack of coordination between the various stakeholders [48]. There are indeed multiple information gaps at all levels, including between the consumer and the healthcare provider (in terms of prescription drug choice), between the healthcare provider and the manufacturer (in terms of the therapeutic qualities of the product), and even between the manufacturer and the regulator. The pharmaceutical system’s vulnerabilities to corruption are many and increasingly understood as a pervasive problem with negative effects on health status and social welfare [9].

Corruption in the pharmaceutical system specifically can compel the global poor, who are the most vulnerable to its worst effects, to make sub-optimal choices that may include purchasing drugs from unqualified or illegal drug sellers to save money, not taking needed medicines if they are unavailable in the public health system, or impoverishing themselves further by having to purchase expensive drugs in the private health system. Further, the transnational criminal trade in substandard/spurious/falsely-labeled/falsified/counterfeit medical products is a pervasive problem in global markets, and is recognized as a global public health threat with severe consequences, including patient death, treatment failure, and possible antimicrobial resistance [49]. Thus, pharmaceutical governance, with a focus on anti-corruption activities, is essential to improve healthcare services and patient outcomes globally.

For decades, global development institutions ignored addressing corruption in their policy and programmatic areas. There are many reasons why this was the case – it is challenging to provide substantial data about its occurrence and its impacts, and it is a highly sensitive and politically charged issue. Thanks to growing public awareness about the deleterious impacts of corruption, particularly in terms of development goals, addressing corruption is now squarely embedded in the global development agenda and it is even included as a specific target within the new SDGs. However, even before these developments, global organizations, donor funded organizations, and civil society, such as the WHO, the Medicines Transparency Alliance, the Global Fund for AIDS, Malaria and Tuberculosis, and most recently, TI, have been active in this area by launching policy and/or operational work on transparency and accountability, two key components of good governance in pharmaceutical systems.

The integrity of the global pharmaceutical supply chain is indispensable to securing health outcomes today and tomorrow [46]. However, as stated above, governance matters. For example, to avoid breaches in the pharmaceutical procurement system, an area particularly vulnerable to corruption, e-procurement should be the norm. Electronic bidding creates a platform through which multiple healthcare facilities can upload their tenders and where prequalified suppliers that have a proven reliability can participate. Open contracting, along with e-procurement, can help improve transparency and accountability in the procurement process and ideally lead to financial savings as well as more assurance that good quality medicines are being procured [50]. Making the invisible visible and ensuring that mechanisms for good governance that promote transparency and accountability are in place, not just in procurement but in all areas of the pharmaceutical system, are important for improving global pharmaceutical access to good quality and essential medicines and to achieve health gains.

## Health security and corruption

### Joshua Michaud (Fig. 9)

**Fig. 9:**
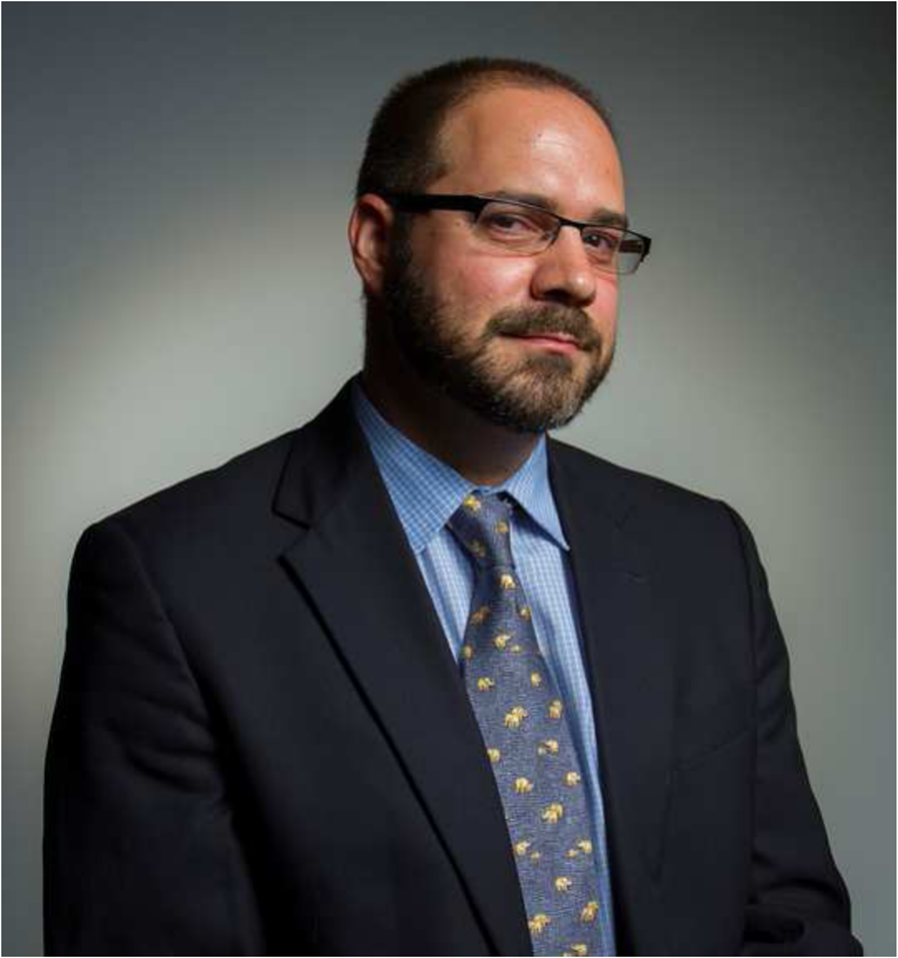
Joshua Michaud is an Associate Director for Global Health Policy at the Kaiser Family Foundation, and an authority on global health policy issues such as financing, the role of US agencies in global health, global health diplomacy, and health security and emerging diseases. He is also a Professorial Lecturer at the Johns Hopkins University School of Advanced International Studies (SAIS) in Washington DC, where he teaches courses on global health policy and health and development

We live in an age of epidemics and potential pandemics. One need only list some of the key threats from the headlines of the last few years alone to get a sense – Zika, Ebola, MERS, influenza, and rising antimicrobial resistance. Above and beyond the morbidity and mortality they cause, these events often carry huge economic and social disruption costs, and therefore are increasingly seen not just as public health problems, but also as national and global security concerns [51].

Health security efforts, which have received greater attention and funding from policymakers in the last several years, seek to minimize vulnerability to these types of threats. While the increased attention is welcome, all parties must recognize that such efforts are vulnerable to corruption just as with other areas of healthcare. As previously discussed, corruption can take many forms: from “petty” corruption such as absenteeism or bribe-taking, to criminal activity such as theft and embezzlement of funds, to poor governance and lack of compliance with rules and regulations abetted by nepotism and non-merit-based hiring practices [52]. Corrupt practices not only impact individual patients and localities where they occur, but in the case of emerging diseases, they can potentially have more widespread, even global, consequences for human health and welfare.

As outlined in the newly launched Global Health Security Agenda (GHSA), the aim of health security efforts is to help countries build a set of core capabilities to prevent, detect, and respond to emerging health crises. However, even if GHSA documents do not mention corruption specifically, these capacity building efforts are vulnerable just like any other public health initiatives. The remainder of this section will briefly discuss examples of corruption that can jeopardize capabilities in each of the three focus areas of the GHSA.

### Prevent

Preventing an outbreak from occurring in the first place is the best possible health security outcome, but requires an effective public health system with good governance and oversight being in place. Unfortunately, many healthcare systems struggle with providing access and high quality services, often due to a variety of corrupt practices [53–55]. Efforts to stem the spread of antimicrobial resistance – one of the key GHSA areas of prevention effort – are jeopardized by the infiltration of poor quality, falsified, substandard, and counterfeit medicines, including antimalarials and antibiotics, into pharmaceutical supply chains [46, 47, 56].

Health security also requires empowered, effective leadership and oversight, but the system of global health governance has been weakened by placements in key positions based on politics and personal connections rather than expertise or effectiveness. As an example, WHO country representatives in West Africa at the time of the 2014 Ebola outbreak were “politically motivated appointments” whose actions were viewed as ineffective, and even a hindrance, during the early response to the disease [57–59]. Corruption reportedly plagues the selection of member state delegations and the process of electing WHO leadership [60]. We are at an important juncture in this regard, as member states have already begun negotiations for selecting the next Director General of the WHO, a process that has been characterized as far from open and transparent.

### Detect

Early detection of emerging disease events is critical for intervening quickly to stem impacts, and detection relies on robust surveillance systems with a motivated and effective workforce at its foundation. Astute observation by local health practitioners is often the first step in early detection of an outbreak. It is unfortunate, then, that many communities often lack trust in their local health providers due to corrupt practices such as requirements to pay bribes for services even when nominally free and high rates of chronic absenteeism among health workers. This was certainly a factor in the Ebola epidemic; in 2013, 48 % of patients in Sierra Leone and 40 % in Liberia reported paying bribes to access health services [7] and mistrust between local communities and primary public healthcare providers in Sierra Leone pre-existed the outbreak [61, 62].

Even after an outbreak is detected, reporting by authorities can be incomplete or delayed due to self-interest and skewed incentives. The SARS episode in China provides an example where ability to intervene early was undermined by conscious misrepresentation of information in order to protect individuals’ careers and the government’s reputation [63]. Similar behavior has been noted in Saudi Arabia and South Korea regarding MERS, and in Venezuela regarding Zika [64–66].

Utilizing a broader, more decentralized, and technology-driven approach to surveillance can help address some of these challenges. For example, linking mobile phone disease reporting from civil society and private sector sources to formal networks can democratize surveillance and loosen central authorities’ tight control over critical outbreak information [67]. Robust platforms already exist for this more informal, non-centralized type of reporting, though not without their own challenges [68, 69]. This has already occurred to a limited extent at the global level, as the most recent revision of international regulations around disease reporting allow WHO, for the first time, access to and use of information from non-governmental sources for the purposes of identifying outbreaks of concern [70].

### Respond

Epidemic response can involve many actors and new funds pouring in, sometimes without adequate oversight and controls being in place. Injecting funds into weak systems not ready to absorb them or track them can be a recipe for crimes of opportunity like embezzlement and diversion of resources for private gain, as emergency responses in countries of all income levels have demonstrated [71–73]. In the case of Ebola, Sierra Leone’s auditor-general found that one-third of the country’s own contributions to the response within its borders was unaccounted for [74], while Liberia’s General Auditing Commission found numerous financial and reporting irregularities in Ebola response money in the country [75]. Further, Saudi Arabia’s government reported US$ 266 million of its funding for MERS to have been used in a corrupt manner [76].

To combat such diversion of funds there is no substitute for vigilance and having robust, risk-based approaches in place prior to the occurrence of an outbreak. This means having policies, procedures and the means to provide due diligence for recipients of funds, plus proper documentation, reporting, monitoring, and oversight of funding. Finally, transparency on aid flows, covering public and private actors, can help provide more accountability during an outbreak [77].

These are only a few examples of how corruption can impact health security, and what can be done to address it. The only way to truly and sustainably address emerging threats is to ensure all corners of the globe have a minimum level of public health capacity, and a functioning system of governance is a key part of this goal that is not always emphasized. Through the GHSA and other initiatives, efforts are now underway to bolster public health capabilities; however, accountability and oversight mechanisms to combat corruption should be considered, as these will ultimately help make funds go even farther and save even more lives.

## Anti-corruption and the SDGs – a pathway forward

### Taryn Vian (Fig. 10)

**Fig. 10:**
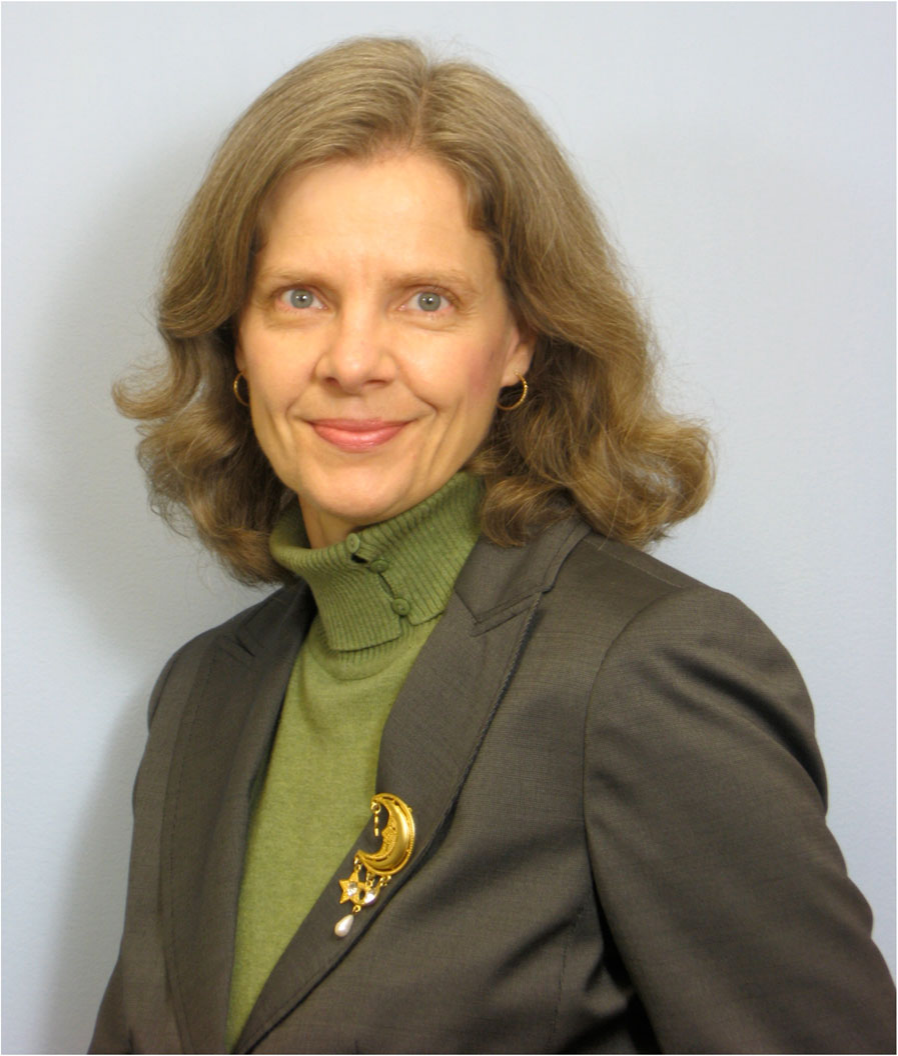
Taryn Vian is Associate Chair of Education and Associate Professor of Global Health at the Boston University School of Public Health. Her research and teaching focus on corruption and health, good governance, financial reforms, and management systems. She has analyzed corruption vulnerabilities in various countries for clients including USAID, Transparency International, the Council of Europe, and WHO. She has participated in Global Advisory Groups and expert meetings organized by the Bill and Melinda Gates Foundation, USAID, UNDESA, and WHO on public engagement in anti-corruption, transparency in the pharmaceutical sector, and supply management

Building on the momentum created by the Millennium Development Goals, the SDGs have set an agenda to eradicate poverty, promote peace, protect the environment, and advance population well-being over the next 15 years [78]. SDG 3 (“Ensure healthy lives and promote well-being for all at all ages”) includes targets to reduce mortality, end epidemics, manage non-communicable diseases, and achieve systems-wide improvements in access and financing, among others [79].

The SDG goals and targets also include a commitment to improve governance. Strong institutions and good governance are essential to ensuring equitable access to quality public services, including health and education [80]. With the SDGs, we can expect to see more resources dedicated to strengthening institutions and building capacity to improve governance. This is an important opportunity to invest in health systems strengthening to prevent and control corruption.

SDG 16 (“Promote just, peaceful and inclusive societies”) specifically includes a sub-target to “substantially reduce corruption and bribery in all their forms”. The UN Inter-Agency Expert Group on SDG Indicators proposes to measure this target by the ‘*percentage of persons who had at least one contact with a public official, who paid a bribe to a public official, or were asked for a bribe by these public officials, in the previous 12 months, disaggregated by age group, sex, region and population group*’ [81].

While a single target cannot capture the myriad forms of corruption, the health sector provides many opportunities for bribes or informal payments, especially within the procurement process, during health inspections, and in interactions between individuals and clinicians. For example, over 30 % of respondents from eight African countries reported having to pay bribes to access healthcare services in one study, with the poorest being most disadvantaged [82]. A review of audit reports for health grants in Brazil found that 55.9 % of municipalities had experienced at least one incident of corruption, including procurement fraud and over-invoicing [83]. Looking forward, health sector leaders should be setting their own intermediate targets to reduce opportunities and incentives for bribes and informal payments in order to achieve the SDGs.

Some strategies are known to work. Informal payments can be reduced by making sure patients are aware of official pricing policies, implementing payment systems reforms, and improving incentives of healthcare professionals to provide good quality care so that patients do not need to resort to bribes [84, 85]. Bribes in procurement can be controlled through price monitoring to detect and investigate procurements which may have inflated prices to conceal bribes, through electronic procurement systems which control discretion and increase transparency, and by regular internal and external audits [86, 87]. Community monitoring for accountability has proven effective in reducing medicine stock-outs, unjustified absenteeism, informal payments, and other forms of abuse of power [88]. These strategies need to be adapted to context, paying attention to local knowledge and building on local values that are compatible with improved integrity and better governance.

Researchers studying health sector corruption in Europe developed a typology of six common corruption problems, including bribery in medical service delivery, procurement corruption, improper medical device and medicines marketing relations, misuse of (high) level positions, undue reimbursement claims, and fraud and embezzlement of medicines and medical devices [89]. Yet, the prevalence and patterns of these problems vary by country. Reflecting these differences, priority-setting for anti-corruption depends, in part, on the financing system in place – corruption risks in tax-based systems generally include diversion of funds at the ministerial level, informal payments, corruption in procurement, and abuses affecting quality of care, while in social insurance systems there are higher risks for corruption due to excessive treatment, billing fraud, and diversion of funds [89].

Analyzing risks in particular settings is important, and can draw on analysis of household budget survey data (to detect informal payments), medicine price surveys (to detect excessive payments for commodities which might indicate bribery or bid-rigging), past audit reports (to detect gaps in financial controls), and interviews with key informants (to identify areas where excess discretion or other systems weaknesses may lead to abuses) [90–92]. The effectiveness of interventions will depend as much on a country’s culture, history, institutional constraints, and capacities as it does on analysis of forms of corruption. Attempting to apply standardized solutions without concern for the particular corruption problem in its own context is counterproductive.

We can strengthen governance in the health sector, and this will help countries to achieve the SDGs. Monitoring bribery (the target for SDG 16) through health sector surveys will help focus attention on the problem, but it is not a solution. We need to train a new generation of health leaders who can diagnose health sector corruption risks and incorporate solutions into health policies and plans. Unaddressed corruption directly impacts attainment of the SDG health goals, and cannot be accepted.
